# (Re)weaving intimacies with ‘Āina for our past, present, and future

**DOI:** 10.3389/fpubh.2026.1842672

**Published:** 2026-06-26

**Authors:** Caleb Rivera, Māhealani Taitague-Laforga, Joie Agard, Kūlani DeSimone, V. Lu‘ukia Nakanelua, Mapuana C. K. Antonio

**Affiliations:** 1 Department of Psychology, University of Hawai‘i at Mānoa, Honolulu, HI, United States; 2 Native Hawaiian and Indigenous Health, Department of Public Health Sciences, University of Hawai‘i at Mānoa, Honolulu, HI, United States; 3 Department of Quantitative Health Sciences, University of Hawai‘i at Mānoa, Honolulu, HI, United States

**Keywords:** epistemology, Indigenous, land, native, wellbeing

## Abstract

Colonial systems have sought to sever Kanaka ‘$\bar{\text{O}}$iwi (Native Hawaiian) from ‘āina (land, that which feeds), undermining Indigenous flourishing and community wellness. Yet, the ‘ōlelo no‘eau (Hawaiian proverb) reminds us, i ka wā ma mua, ka wā ma hope, the future is in the past. This paper traces the journey of ‘āina relationships across time: from ancestral epistemological foundations, through colonial disruption, to contemporary revitalization, and toward futures grounded in collective autonomy, dignity, and liberation. ‘$\bar{\text{A}}$ina represents far more than physical territory; it encompasses a living, reciprocal relationship between people and place that sustains Kanaka ‘$\bar{\text{O}}$iwi for generations. Drawing on mo‘olelo (history, lore), ‘ōlelo no‘eau, mele (chants, songs), and scholarship, we examine how knowledge of ‘āina emerges through relational and genealogical frameworks, embodied learning, and place-based practices. We unravel how colonization systematically disrupted these relationships while highlighting contemporary initiatives that are (re)claiming and (re)vitalizing connections to ‘āina. We intentionally close by (re)weaving intimacies forward, envisioning what becomes possible when Kanaka ‘$\bar{\text{O}}$iwi epistemologies are honored, when land and water are returned back to community, and when wellness is not about resilience to ongoing harm but thriving within restored intimacies with ‘āina.

## Introduction

Through a Kanaka ‘Ōiwi (Native Hawaiian)^1^ worldview, ‘āina (land, that which feeds) embodies living ancestors whose presence is imperative to the health and wellbeing of all that exists across generations (1). Mele (chants, songs), and mo‘olelo (history, lore) recognize ‘āina as the physical, psychological, and spiritual foundations of Kanaka ‘Ōiwi culture (2, 3). While Western perspectives reduce land to economic assets or natural resources to be exploited, Kanaka ‘Ōiwi understandings of ‘āina transcend notions of land, encompassing a dynamic, reciprocal relationship between people and place. As expressed in the ‘ōlelo no‘eau (Hawaiian proverb), “Hānau ka ‘āina, hānau ke ali‘i, hānau ke kanaka” (Born was the land, born were the chiefs, born were the common people) (4). This relationship positions Kanaka ‘Ōiwi not as owners but as caretakers with crucial responsibilities toward the land that sustains them.

These relationships constitute what Osorio (5) describes as Indigenous intimacies. Intimacies are the deeply personal, embodied, and reciprocal connections between Kanaka ‘Ōiwi, ‘āina, and ancestors that form the foundation of mauli ola (optimal health and wellbeing). Unlike Western framings that often reduce Indigenous-land relationships to political nationalism or resource dependency, intimacies capture the affective, spiritual, and genealogical dimensions of aloha ‘āina (love of the land) that sustain Kanaka ‘Ōiwi for generations. These deep intimacies are captured in contemporary Kanaka ‘Ōiwi health and wellbeing frameworks like the Lokahi triangle, Kūkulu Kumuhana, and Ke Ao Nōweo ‘Ula among others (6–8), situating the connection to ‘āina as integral to one's mauli ola.

This paper unravels intimacies with ‘āina across time, tracing a journey from epistemological foundations and traditional practices, through colonial disruption, to contemporary revitalization efforts. Rather than ending with recommendations, we close by (re)weaving intimacies forward. Throughout, we engage mo‘olelo, ‘ōlelo no‘eau, mele, and scholarship as relatives. Within Kanaka ‘Ōiwi epistemology, mo‘okū‘auhau (genealogy) functions methodologically as a blueprint, organizing knowledge genealogically and locating us within the relationships we write about. We attend simultaneously to mind, body, and spirit as ways of knowing. We read mele for the environmental, political, and spiritual knowledge carried in their cadence and kaona (hidden meaning). We receive mo‘olelo as epistemological grounding that records our ancestral relationships, ecological knowledge, and cosmological truths, returning to them across different wā (eras) and letting their teachings shape our interpretations. We sit with ‘ōlelo no‘eau as ancestral intelligence, layered teachings that guide contemporary thought. We prioritize the scholarship of Kanaka ‘Ōiwi and other Indigenous scholars as extensions of these same mo‘okū‘auhau, recognizing that citation itself is a methodological practice that honors Indigenous intellect and truths. The organizing ‘ōlelo no‘eau, “I ka wā ma mua, ka wā ma hope” (The future is in the past) (9), is itself methodology. We walk backward into the future, allowing ancestral knowledge to shape our analysis.

### Epistemological foundations of Kanaka ‘Ōiwi knowledge

Kanaka ‘Ōiwi epistemology is grounded in the understanding that knowledge emerges from relationships, shaping how ‘āina is regarded as a relative within reciprocal systems of care and responsibility, rather than a mere resource (10). Expanding on this, Oliveira (11) illustrates how ancestral Kanaka ‘Ōiwi relationships with place are cultivated through active engagement, where practices such as mele, mo‘olelo, linguistic nuance, and hula function as relational and cartographic expressions of knowledge and ‘āina. Antonio et al. (12) demonstrate that wellbeing in a Kanaka ‘Ōiwi worldview is achieved by maintaining pono (righteous) relations with ‘āina and the spiritual realm, positioning ‘āina as an active participant in shaping how Kanaka ‘Ōiwi understand, engage with, and care for the land.

This relational understanding is structured through mo‘okū‘auhau, which serves as a fundamental epistemological framework in Kanaka ‘Ōiwi knowledge systems. Rather than organizing knowledge taxonomically or hierarchically, as is common in Western traditions, Kanaka ‘Ōiwi epistemology organizes knowledge genealogically, tracing connections through ancestral lineages that include not only humans but also the more-than-living-world (9, 13). The Kumulipo (Hawaiian creation chant), which spans over 2,000 lines and 16 wā, exemplifies such mo‘okū‘auhau by detailing the births of all living things, with humans emerging late in the sequence, preceded by coral, marine life, plants, and other more-than-living-world beings (14). This positions humans as younger siblings to ‘āina, establishing a natural kuleana (privilege and responsibility) to observe, listen, and learn from them.

Mo‘olelo and mele serve as vehicles through which Kanaka ‘Ōiwi epistemology transmits and preserves knowledge about ‘āina (15, 16). The mo‘olelo of Hāloa (the first Kanaka ‘Ōiwi) is a prime example. This mo‘olelo recounts how Ho‘ohōkūkalani (goddess of the stars) and Wākea (Sky Father) had a stillborn child, Hāloanakalaukapalili, who was buried on the eastern side of their house. From that burial grew the first kalo plant. Ho‘ohōkūkalani later gave birth to a healthy son named Hāloa, in honor of his older sibling, and Hāloa became the ancestor of all Kanaka ‘Ōiwi people. This mo‘olelo establishes kalo (taro) as the older sibling, providing nourishment for people. As the younger sibling, humans have the kuleana to care for kalo and ‘āina that sustains them. Mele composed for specific ahupua‘a (traditional land divisions) carry knowledge rooted in place, preserving details of rainfall patterns, plant communities, and resource management practices while fostering emotional and spiritual relationships to ‘āina (17, 18). Basham (19) reminds us, “We have used mele to record and recount our histories and stories...Mele have been a vital part of our cultural belief systems and practices, our connection to our ‘āina…as well as our formal religious practices and our informal daily practices. Mele have also been vital to our political theories, ideas, and practices.” Together, mele and mo‘olelo form inseparable epistemological foundations, where meaning is layered, contextual, and deeply relational (20). Saffery (21) urges Kanaka ‘Ōiwi to revive these knowledge forms, as they encode environmental wisdom and are tools for resistance and resurgence.

### Traditional practices and systems of ‘āina stewardship

To fully comprehend the struggles Kanaka ‘Ōiwi encounter, while (re)establishing pathways to mauli ola through relationships with ‘āina, one must understand Kanaka ‘Ōiwi epistemologies that preceded western contact. Beginning with the kapu system (taboo, prohibition), this social, economic, and political system governed human interactions with ‘āina through integrated religious and practical dimensions. According to Else (22), the kapu system established three primary types of relationship interactions: among social classes, between people and gods, and between people and nature. The third interaction, between people and nature, is what we now recognize as ecological management principles. The kapu system established specific periods for planting during kau (the temperate dry season, associated with summer) and for harvesting during ho‘oilo (the temperate wet season, associated with winter), creating a systematic approach to resource utilization that aligned human activity with natural cycles (23). This intricate system reveals how Kanaka ‘Ōiwi regulated human-‘āina relationships through protocols.

The ahupua‘a system represents perhaps the most comprehensive expression of Kanaka ‘Ōiwi understanding of ‘āina as an integrated whole (see Supplementary material C). Traditionally extending from uka (upland) to kai (ocean), ahupua‘a were watershed-based land divisions that functioned as self-sustaining ecological and social units within a larger hierarchical structure of land organization (24). Each ahupua‘a was given a name, often reflecting a distinctive characteristic, resource, or historical connection to that place, reinforcing the place-based knowledge systems discussed earlier.

Within these divisions, land was categorized into zones based on elevation, biological communities, and human use. The upper watershed forests known as wao akua (realm of the gods or spirits) were recognized as sacred spaces crucial for water capture, with limited human disturbance permitted (25). The middle zones contained wao kanaka (realm of humans), where agricultural activities were concentrated, while the lower coastal zones supported fishing communities and aquaculture systems. Each ahupua‘a was governed by stewards. Konohiki (headman of an ahupua‘a) oversaw resource allocation and the enforcement of the kapu system, while day-to-day management decisions were often made by maka‘āinana (commoners) who possessed intimate and unique knowledge of environmental conditions (26). This governance system ensured resources were managed sustainably, while preserving for future generations.

Within ahupua‘a systems, Kanaka ‘Ōiwi developed sophisticated agriculture adapted to Hawai‘i's environment. Among these innovations was lo‘i kalo (see Supplementary material D), a system created to grow taro in wetland terraces. Beyond agricultural purposes, lo‘i kalo held profound cultural significance derived from the mo‘olelo of Hāloa, connecting farmers directly to their ancestral lineage through cultivation of their older sibling (27). In drier areas (more upland), Kanaka ‘Ōiwi developed dryland farming systems like taro cultivation and māla (cultivated field) for plants such as ‘uala (sweet potato), mai‘a (banana), ‘ulu (breadfruit), and many other crops (28). Techniques such as mulching with stone pavements, creating windbreaks, and timing planting to seasonal rainfall and moon phases demonstrate the sophisticated ecological knowledge embedded in these practices (29, 30). The loko i‘a (cultivated Hawaiian fish ponds), between 10 and 100 acres in size, represents another impressive system (see Supplementary material E). These engineered aquacultural systems transformed coastal areas into productive food systems that enhanced natural ecological processes (31), which provided sustenance for humans and the more-than-living-world in each ahupua‘a.

Physical practices of ‘āina stewardship were inseparable from spiritual dimensions. This intimate connection began at birth, when families planted a newborn's piko (umbilical cord, center) or ‘iewe (placenta) in the earth, symbolically linking the child to their ancestral lands (32). At the end of one's life, the body would return to the ‘āina, completing a spiritual cycle that connected human existence to place across generations (33). Between these life transitions, Kanaka ‘Ōiwi maintained their spiritual relationship with ‘āina through daily and seasonal practices. For instance, major agricultural and fishing activities began with ceremonial offerings to honor deities and elements such as Lono (akua of the Makahiki season) for agriculture and Kanaloa (akua of the ocean) and Kū‘ula (fish akua) for water resources (34). The annual Makahiki (Hawaiian new year) festival season highlighted this integration, honoring Lono while establishing periods of rest (35). During this 4-month season, communities engaged in ritualized processions where the image of Lono would circuit the island, stopping at each ahupua‘a in a practice that connected spiritual obligation with resource management (36).

Sustaining spiritual practices required intergenerational knowledge transfer rooted in everyday life. For example, children learned their role and responsibility to ‘āina by working alongside ‘ohana (family), which gradually led to greater responsibilities as they demonstrated readiness (37). Adults engaged in more formal apprenticeships, cultivating their knowledge of the ‘āina through fishing, healing, and other cultural practices. Together, these traditional practices created an integrated system of ‘āina stewardship that maintained ecological health and wealth. By approaching land as ancestor and provider, Kanaka ‘Ōiwi developed sustainable relationships with their environment that supported substantial populations while balancing biodiversity and ecosystem function over centuries. What took generations to build, colonial forces would disrupt within decades.

### Impacts of colonization on ‘āina relationships

One of the most devastating impacts of colonialism was the introduction of foreign diseases, which decimated the Kanaka ‘Ōiwi population from an estimated pre-contact population of 800,000–1,000,000 to around 40,000 by 1893 (38). This population collapse disrupted the transmission of intergenerational knowledge critical to relationships with ‘āina. The breaking of the kapu system in 1819, known as ‘ai noa (free eating), marked another pivotal disruption in traditional Kanaka ‘Ōiwi relationships with ‘āina (23). The erosion of ceremonial cycles that aligned human activities with natural rhythms disrupted agricultural calendars, weakening ecological knowledge embedded in seasonal practices (22). This shift, occurring months before the arrival of missionaries in 1820, created a spiritual and governance void, which were eventually filled by Western religious and economic systems.

One of the most consequential legal restructurings of ‘āina relationships occurred through the Māhele (land division) of 1848, which fundamentally altered traditional land tenure systems by introducing Western concepts of private property (39). This process transformed land from a collective resource held in trust to a commodity that could be bought, sold, and owned exclusively. Although initially intended to protect Kanaka ‘Ōiwi land rights, the Kuleana Act ultimately had the opposite effect, enabling foreign interests to acquire vast parcels of Kanaka ‘Ōiwi ancestral lands (9). This privatization process severed the revered ahupua‘a system by fragmenting watersheds into disconnected parcels with incompatible land uses. Where traditional ahupua‘a management required collaboration and coordination across ecological zones, private property rights emphasized individual decision-making.

The rise of the sugar plantation industry around 1848 exacerbated the ecocide of the ‘āina (40). Plantation operations diverted massive quantities of water from traditional agricultural regions, leaving many lo‘i kalo without sufficient resources to maintain production (41). By the 1920s, an extensive network of water infrastructure, including ditches, tunnels, and sluiceways, was redirecting more than 800 million gallons daily from natural waterways and mountain sources to irrigate sugar plantations (42). This massive hydrological reengineering prioritized commercial exports over subsistence agriculture, systematically undermining traditional food systems while increasing dependency on imported goods.

### Overthrow of the Hawaiian Kingdom and changes to water/land law

Prior to the overthrow, Kanaka ‘Ōiwi leaders inscribed genealogical relationships into Kingdom law. The 1840 Constitution affirmed that ‘āina “belonged to the chiefs and people in common” (43), codifying the reciprocal bonds between mō‘ī (king, queen), ali‘i (chief), maka‘āinana, and ‘āina. This legal design was eventually dismantled through the illegal overthrow of the Hawaiian Kingdom in 1893, orchestrated by American businessmen with the support of United States military forces (44). Following the overthrow, approximately 1.8 million acres of Crown and Government lands were seized and eventually transferred to U.S. control through the annexation in 1898 (45, 46). As Trask articulates, this dispossession ruptured the familial relationship with ‘āina, shifting land management from Kanaka ‘Ōiwi cultural values to profit-driven interests (47).

This political dispossession was compounded by cultural dispossession through the 1896 law mandating that English be the official language of education, essentially defunding and devaluing the Hawaiian language as a result (48). By removing the primary vehicle through which place-based and overall knowledge was transmitted, this final colonial intervention led to the comprehensive disruption of Kanaka ‘Ōiwi relationships with ‘āina (49).

### Contemporary revitalization of ‘āina relationships

Kanaka ‘Ōiwi have demonstrated remarkable resilience in maintaining and revitalizing relationships with ‘āina through initiatives that reclaim traditional knowledge while adapting to contemporary contexts. Community-based resource management has emerged as a powerful vehicle for ‘āina reconnection, particularly through traditional food system restoration. Recent ‘Ōiwi organizations (50–54) continue to restore and revitalize ancient fishponds across Hawaiian islands, combining traditional ecological knowledge with contemporary science to create systems that address food sovereignty while restoring ecological function. Similarly, lo‘i kalo restoration efforts (55–60) have reclaimed agricultural lands while (re)establishing traditional water flow pathways that benefit entire ahupua‘a systems. These efforts often face obstacles including limited land access, water rights disputes, and funding models that fail to recognize Indigenous definitions of impact (27, 61–63). Their continued growth reflects the resilience of both the knowledge systems and the communities sustaining them.

Educational initiatives have simultaneously revitalized ‘āina relationships by transmitting place-based knowledge to new generations. Hawaiian language immersion schools are creating environments where students learn not only ‘ōlelo Hawai‘i (Hawaiian language) but also the environmental knowledge layered within it (49). Beyond formal education settings, community organizations have pioneered equally impactful approaches. Ho‘āla ‘$\bar{\text{A}}$ina Kūpono, for instance, engages community members of all ages in Indigenous Culture-as-Health initiatives through afterschool programs and seasonal activities centered on ‘āina.

Legal movements and ‘āina back initiatives have worked to secure land and water rights necessary for these revitalization efforts to flourish. Tanigawa Lum shares her analysis of the Wai‘oli Valley Taro Hui's efforts to protect water rights, these legal battles are not solely about resource access but represent a broader movement toward restorative environmental justice that centers ‘āina and Indigenous knowledge systems in opposition to Western legal frameworks (64).

## Discussion

The epistemological foundations, traditional practices, colonial disruptions, and contemporary revitalization efforts discussed thus far can be understood through the Nā Pou Kihi framework developed by Kaholokula that conceptualizes the determinants of Kanaka ‘Ōiwi health and wellbeing (65). This framework uses the metaphor of four corner posts (pou kihi) that together support a hale (house): Ke Ao ‘Ōiwi (the native world), Ka Mālama ‘$\bar{\text{A}}$ina (the caring of the land), Ka Hana Pono (the right behaviors), and Ka Wai Ola (the life-giving waters). Overall, this framework expounds on how ‘āina relationships are not supplemental to Kanaka ‘Ōiwi wellbeing but foundational to it.

(Re)weaving intimacies forward requires us to co-create a future grounded in collective healing to achieve balance. We envision intimacies that move beyond restoration toward true embodiment of mo‘olelo as markers of health and wellbeing, while honoring the inherent hierarchy that exists for us, as people, in relation to ‘āina and akua (elements, gods). Our ability to embody ‘ōlelo Hawai‘i and mo‘olelo becomes the act of health and wellbeing itself. To know our mo‘okū‘auhau intimately demonstrates the depth of our pilina (relationship) to ‘āina, to each other, and to pō (darkness). This is a future where sustainability is centered in our daily lives, with a return to piko, where cultural knowledge is lived.

With our grounding in ku‘una ‘ike Hawai‘i (Hawaiian knowledge & practices), we envision a future where health is reflected in the thriving ‘āina that surrounds us, in the abundance of wai (fresh water) flowing as it once did, in native birds chirping as signs of life as they traverse Moananuiākea (the Pacific). We dream of a world where the living waters of Kāne (akua of forces of nature that give us life) are restored and free-flowing, where we no longer have to search for the manifestations of Kāne, but can see, feel, and immerse ourselves in its mana (divine power, authority) each day. A world where we, and the countless generations that follow, sustain an active relationship with wai, reflected in the vitality of our lands and the flourishing of our people.

We dream of a future which acknowledges the kauhale system (group of houses comprising a Hawaiian home) not only as functional and practical, but as the ideal arrangement for Kanaka ‘Ōiwi health and wellbeing. This is a future that values collectivism and interdependence over individualism and capitalistic greed. We dream of a future where our ‘ohana remain stewards of ‘āina that belonged to our ancestors. This is a future where keiki (children) and kupuna (ancestors) engage in intergenerational knowledge transfer through mo‘olelo. We dream of a future abundant in ke ao ‘ōiwi as public gathering places for sharing ‘ike kupuna (ancestral knowledge) and engaging in mana exchange.

We dream of a world where “LANDBACK” is no longer a movement or a demand for justice, but a lived reality, one in which our ‘āina, wai, and mana have been fully restored. We envision intimacies restored where jurisdiction over ‘āina returns to itself and its caretakers. We (re)weave a future abundant with pu‘uhonua (sanctuary), where all can thrive without fear of injustice against their ‘āina, their ‘ohana, or their lāhui (nation). Within these restored intimacies, we are not resilient. We are simply well.

## Data Availability

The original contributions presented in the study are included in the article/Supplementary material, further inquiries can be directed to the corresponding author.

## Footnotes

1 We use the term Kanaka ‘Ōiwi throughout this paper to mean Native Hawaiian. This term refers to any person(s) with an ancestral connection, a tie to Hawai‘i carried in their bones (iwi) and passed through generations. This is without reference to blood quantum.
