# Supplementary material for: (Re)weaving intimacies with ‘Āina for our past, present, and future
Source: Front Public Health. 2026 Jun 26;14:1842672. doi: 10.3389/fpubh.2026.1842672 (PMC13350168; doi:10.3389/fpubh.2026.1842672)
Supplement: Supplementary file 1 [file Data_Sheet_1.pdf]

Supplementary Material A: Positionalities of Authors

Caleb Rivera, MA is a Maui Native and PhD student in Community Psychology at the University of Hawaiʻi at Mānoa. His work centers on culture-as-health and strengthening pilina within communities, with particular attention to Kanaka ʻŌiwi experiences. As an ʻŌiwi story weaver, Caleb works with communities across Hawaiʻi to capture moʻolelo, exploring how ʻāina serves as a mechanism for health, healing, learning, and overall wellbeing.

Māhealani Taitague-Laforga, MPH is a Kanaka ʻŌiwi wahine, born and raised in the pae ʻāina of Hawaiʻi on the mokupuni of Oʻahu. She is an ʻōlapa, a dedicated student of hula, deeply grateful to have been nourished with ʻike kūpuna through mele, ʻoli, and moʻolelo from a young age. She holds a graduate degree in Public Health with a specialization in Native Hawaiian and Indigenous Health from the University of Hawaiʻi at Mānoa. Her scholarship explores the determinants of health across Moananuiākea, and she is committed to advancing Native Hawaiian health, resilience, and environmental restoration while strengthening cultural knowledge for future generations.

Joie Agard, MPH is a Kānaka ʻŌiwi woman from Kalaoa, Hawaiʻi. She has a graduate degree in Public Health with a specialization in Native Hawaiian and Indigenous Health from the University of Hawaiʻi at Mānoa. Guided by experiences from her multigenerational household, her work (un)ravels the intricacies of modern and historical living systems for Kānaka ʻŌiwi as they relate to intergenerational knowledge sharing, determinants of health, and ʻohana wellbeing.

Kūlani DeSimone, BS is a Native Hawaiian wahine, born in California and raised in Hawaiʻi, with ancestral roots in Hawaiʻi, Maui, and predominantly Kauaʻi. She is well-versed in both Western and Native Hawaiian perspectives. Dedicated to serving her community and Native Hawaiian organizations, she is committed to advancing the health and well-being of her people and addressing critical health disparities facing the NHPI community.

V. Luʻukia Nakanelua, Esq is a Kānaka ʻŌiwi from Maui whose legal practice area focuses on Native Hawaiian Rights and Environmental Law. Grounded by her cultural grounding, formal education, and lived experience as a kupu (sprout, offspring) of the aloha ʻāina movement, she works with Hawaiʻi's unique communities to address compelling issues of ʻŌiwi knowledge systems and lifeways including the legal duty to mālama ʻāina (natural and cultural resource stewardship), resilience, and food security. She is a graduate of the University of Hawaiʻi William S. Richardson School of Law with certificates in Native Hawaiian Law and Environmental Law. She is a product of the Pūnana Leo o Maui and Kula Kaiapuni o Pāʻia programs and a graduate of the Kamehameha Schools Maui Campus and the Lewis and Clark College in Portland, Oregon where she earned a bachelor's degree in Environmental Studies.

Mapuana C. K. Antonio, DrPH is a Kanaka ʻŌiwi wahine scholar from Wahiawā, Oʻahu whose positionality is inseparable from her aloha and kuleana to her community and ʻāina. She is an Associate Professor and Head of the Native Hawaiian and Indigenous Health (NHIH) Program at the University of Hawaiʻi at Mānoa. Trained in psychology, public health, community-based translational research, and Indigenous methodologies, she engages in strengths-based, community-based, and culturally-grounded research to advance health, healing, and resilience in Native Hawaiian and Indigenous communities.
