# Supplementary material for: (Re)weaving intimacies with ‘Āina for our past, present, and future
Source: Front Public Health. 2026 Jun 26;14:1842672. doi: 10.3389/fpubh.2026.1842672 (PMC13350168; doi:10.3389/fpubh.2026.1842672)
Supplement: Supplementary file 2 [file Data_Sheet_2.pdf]

***Supplementary Material B: Glossary of Hawaiian words***

**ʻāina** - land, that which feeds

**ʻai noa** - free eating

**ahupuaʻa** - traditional land divisions

**akua** - elements, gods

**aliʻi** - chief

**aloha ʻāina** - love of the land

**hale** - house

**Hāloa** - the first Kanaka ʻŌiwi

**hānau ka ʻāina, hānau ke aliʻi, hānau ke kanaka** - Born was the land, born were the chiefs, born were the common people

**Hoʻohōkūkalani** - goddess of the stars

**hoʻoilo** - the temperate wet season, associated with winter

**i ka wā ma mua, ka wā ma hope** - The future is in the past

**ʻiewe** - placenta

**ʻike Hawaiʻi** - Hawaiian knowledge & practices

**ʻike kūpuna** - ancestral knowledge

**Ka Hana Pono** - the right behaviors

**kai** - ocean

**kalo** - taro

**Ka Mālama ʻĀina** - the caring of the land

**Kanaka ʻŌiwi** - Native Hawaiian

**Kāne** - akua of forces of nature that give us life

**Kanaloa** - akua of the ocean

**kaona** - hidden meanings

**kapu** - taboo, prohibition

**kau** - the temperate dry season, associated with summer

**kauhale** - group of houses comprising a Hawaiian home

**Ka Wai Ola** - the life-giving waters

**Ke Ao ʻŌiwi** - the Native world

**keiki** - children

**konohiki** - headman of an ahupuaʻa

**kuleana** - privilege and responsibility

**Kumulipo** - Hawaiian creation chant

**kūpuna** - ancestors

**Kūʻula** - fish akua

**kuʻuna ʻike Hawaiʻi -** Hawaiian knowledge and practices

**lāhui** - nation

**loko iʻa** - cultivated Hawaiian fish ponds

**Lono** - akua of the Makahiki season

**loʻi kalo** - a system created to grow taro in wetland terraces

**maiʻa** - banana

**Makahiki** - Hawaiian new year

**makaʻāinana** - commoners

**māla** - cultivated field

**mana** - divine power and authority

**mauliola** - optimal health and wellbeing

**mele** - chants & songs

**Māhele of 1848** - land division of 1848

**Moananuiākea** - the Pacific

**mōʻī** - king, queen

**moʻokūʻauhau** - genealogy

**moʻolelo** - history/lore

**ʻohana** - family

**ʻōlelo Hawaiʻi** - Hawaiian language

**ʻōlelo noʻeau** - Hawaiian proverb

**piko** - umbilical cord, center

**pilina** - relationship

**pō** - darkness

**pono** - righteous

**pou kihi** - corner posts

**puʻuhonua** - sanctuary

**uka** - upland

**ʻuala** - sweet potato

**ʻulu** - breadfruit

**wā** - eras

**wai** - fresh water

**Wākea** - sky father

**wao akua** - realm of the gods or spirits

**wao kanaka** - realm of humans
