# Supplementary material for: (Re)weaving intimacies with ‘Āina for our past, present, and future
Source: Front Public Health. 2026 Jun 26;14:1842672. doi: 10.3389/fpubh.2026.1842672 (PMC13350168; doi:10.3389/fpubh.2026.1842672)
Supplement: Supplementary file 4 [file Data_Sheet_4.pdf]

Supplementary Material D: Ka Papa Lo'i 'O Kanewai

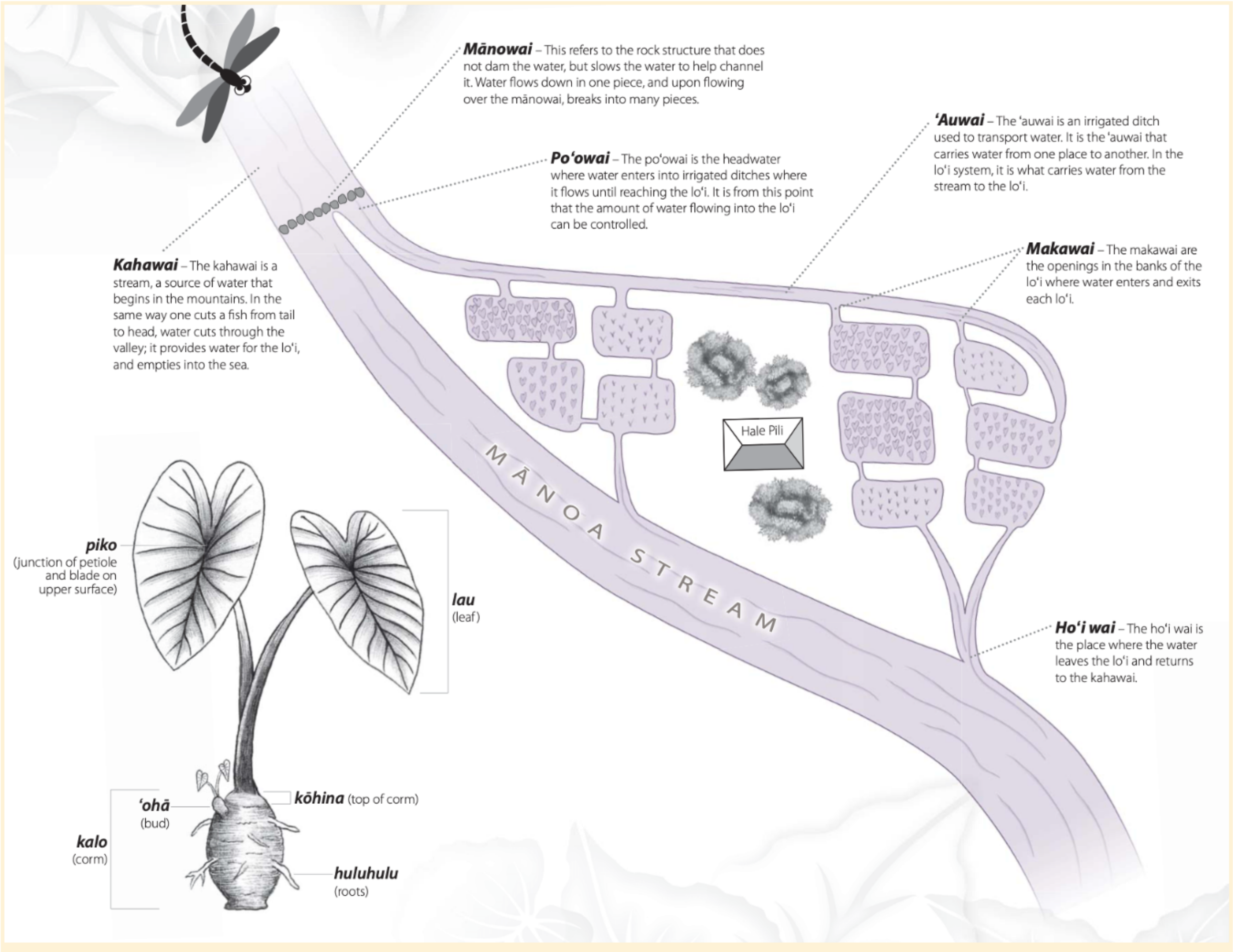

Source: Welina Mānoa (n.d.). https://welinamanoa.wordpress.com/wp-content/uploads/2012/09
