# Supplementary material for: (Re)weaving intimacies with ‘Āina for our past, present, and future
Source: Front Public Health. 2026 Jun 26;14:1842672. doi: 10.3389/fpubh.2026.1842672 (PMC13350168; doi:10.3389/fpubh.2026.1842672)
Supplement: Supplementary file 5 [file Data_Sheet_5.pdf]

Supplementary Material E: Different Types of Loko I'a

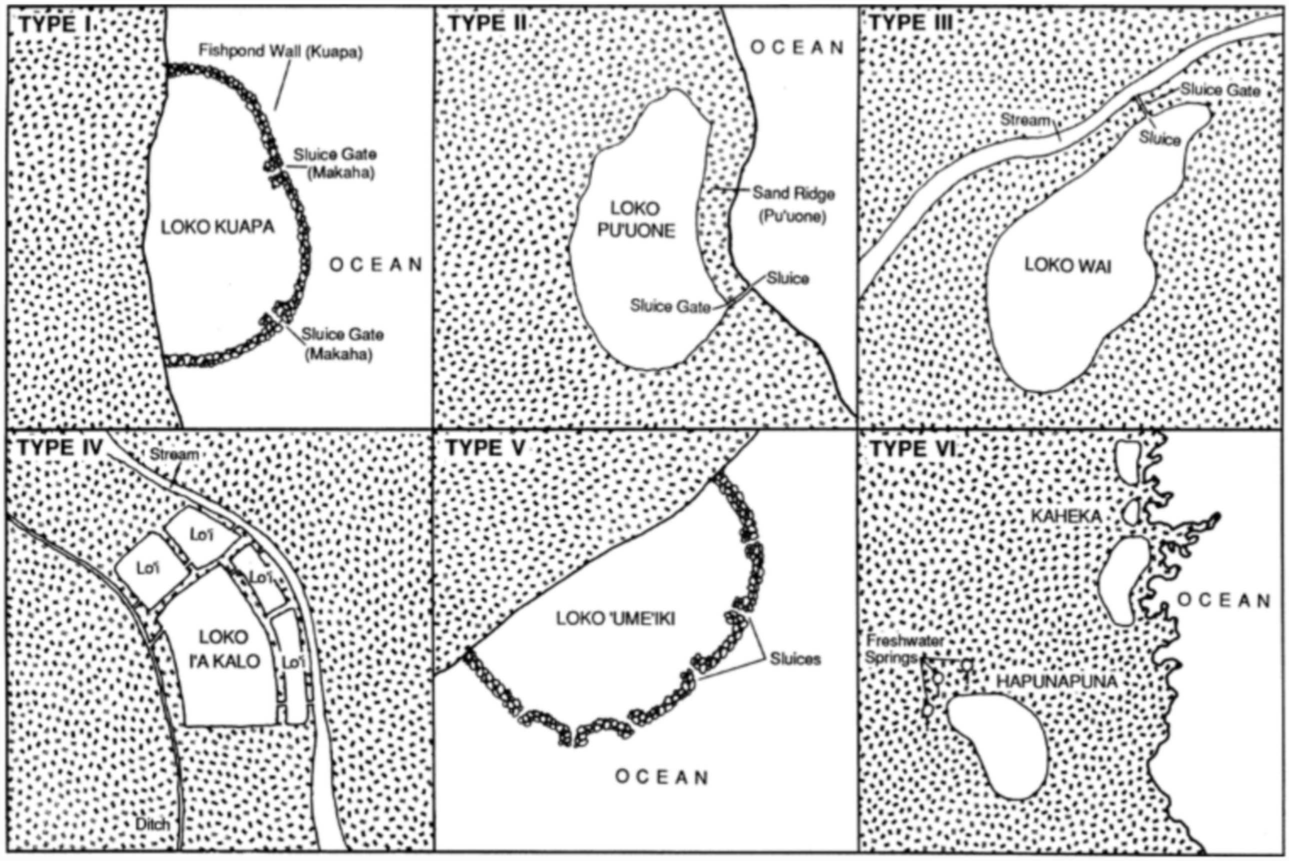

Source: R.A. Apple and William K. Kikuchi, 1975, Ancient Hawaiian shorezone fishponds: An evaluation of survivors for historical preservation, U.S. National Park Service, Department of Interior, p. 157
