# Supplementary material for: (Re)weaving intimacies with ‘Āina for our past, present, and future
Source: Front Public Health. 2026 Jun 26;14:1842672. doi: 10.3389/fpubh.2026.1842672 (PMC13350168; doi:10.3389/fpubh.2026.1842672)
Supplement: Supplementary file 3 [file Data_Sheet_3.pdf]

Supplementary Material C: Pre-Māhele Moku and Ahupuaʻa of Oʻahu

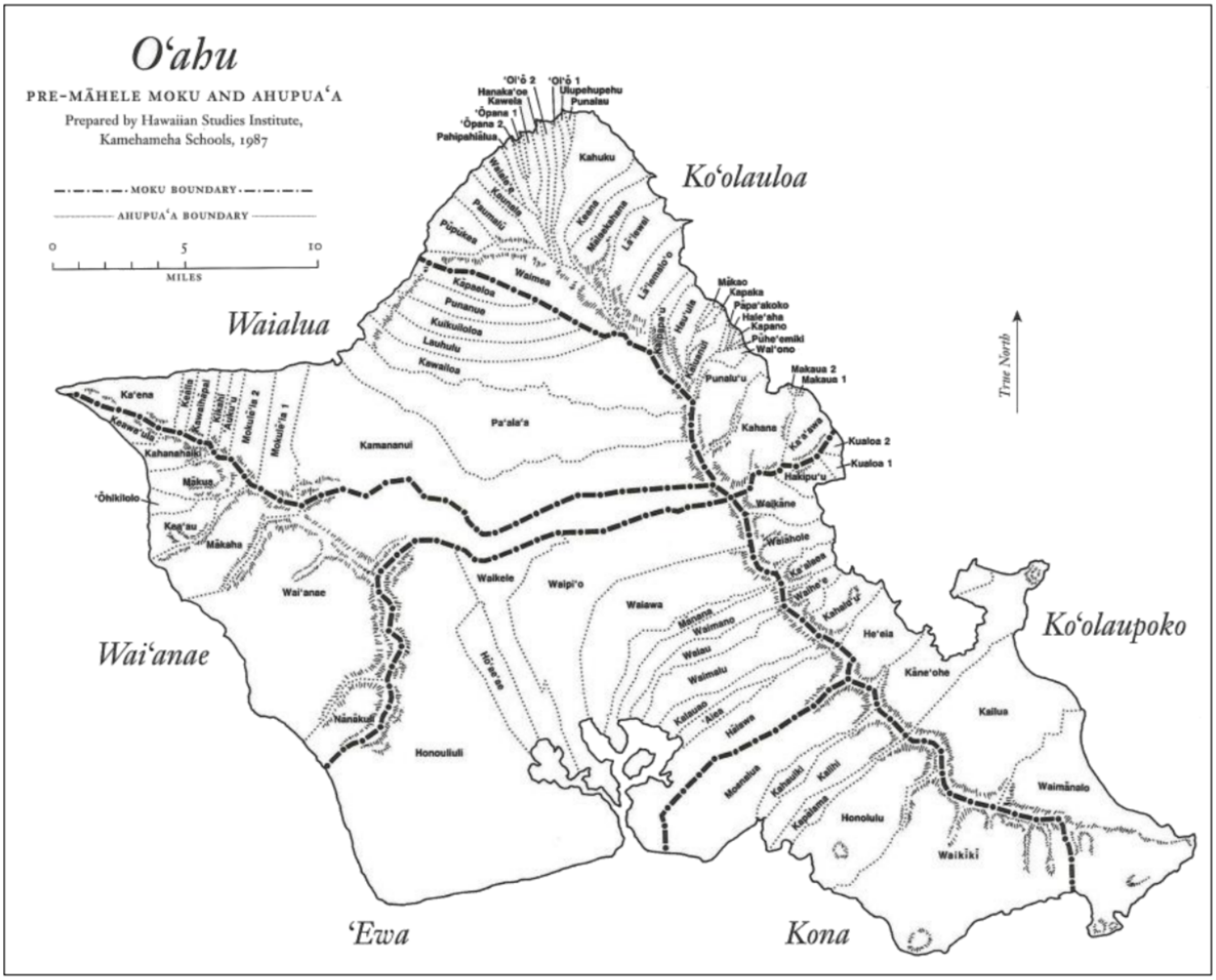

Source: Awa Konohiki. (n.d.). https://avakonohiki.weebly.com/o699ahu.html
